# Paleolithic eyed needles and the evolution of dress

**DOI:** 10.1126/sciadv.adp2887

**Published:** 2024-06-28

**Authors:** Ian Gilligan, Francesco d’Errico, Luc Doyon, Wei Wang, Yaroslav V. Kuzmin

**Affiliations:** ^1^ School of Humanities, The University of Sydney, NSW 2006, Australia.; ^2^ Université de Bordeaux, CNRS, MCC, PACEA, UMR5199, Pessac 33615, France.; ^3^ University of Bergen, Centre for Early Sapiens Behaviour (SapienCE), Department of Archaeology, History, Cultural Studies and Religion, Bergen 5020, Norway.; ^4^ Institute of Cultural Heritage, Shandong University, Qingdao 266237, China.; ^5^ Sobolev Institute of Geology and Mineralogy, Siberian Branch of the Russian Academy of Sciences, Novosibirsk 630090, Russia.

## Abstract

Eyed needles are among the most iconic of Paleolithic artifacts, traditionally seen as rare indicators of prehistoric clothing, particularly tailoring. However, recent finds across Africa and Eurasia show that other technologies like bone awls also facilitated the creation of fitted garments. Nonetheless, the advent of delicate eyed needles suggests a demand for more refined, efficient sewing. This refinement may signify two major developments: the emergence of underwear in layered garment assemblages, and/or a transition in adornment from body modification to decorating clothes, as humans covered themselves more completely for thermal protection. Archaeological evidence for underwear is limited, but the Upper Paleolithic saw an increase in personal ornaments, some sewn onto clothing. Eyed needles may mark a pivotal shift as clothes acquired the social functions of dress, decoupling clothing from climate and ensuring its enduring presence.

## Teaser

Humans invented clothes to cope with past climate changes, then clothes evolved into dress—one clue is the advent of eyed needles.

## INTRODUCTION

The emergence and evolution of clothing was an integral aspect of the culturalization of the human body that has substantially contributed to shaping our human niche and promoted a process of self-domestication (*1*–*4*). Clothing enabled our ancestors to inhabit a wider variety of environments and thereby access different resources and ecological niches (*5*). In its coevolution alongside humans, clothing not only served as a practical necessity for protection and comfort but also became a powerful tool for cultural expression, and social and individual identity (*6*, *7*). Clothing is used by most known human societies to signify group identity, affiliation, and social status. This allowed for the formation of larger and more complex societies, as individuals could identify and cooperate with members of their group, tribe, or community based on shared clothing styles and symbols. The production of clothing, including the selection of materials and tools, required planning and resource management. These skills would have contributed to a more sustainable lifestyle and enhanced the long-term survival and prosperity of human communities. In summary, clothing has played a multifaceted role in human niche construction.

### Paleolithic clothing

Direct archaeological evidence for Paleolithic clothing is elusive. Archaeological findings in Eurasia, dating back to the Early and Middle Pleistocene, indicate the use of stone tools—notably hide-scrapers—to prepare animal skins for thermal insulation as hominins occupied colder environments (*5*, *8*). Evidence for animal skinning and hide scraping has been identified at various sites such as Hoxne (*9*), Qesem Cave (*10*), and Schöningen (*11*, *12*). Bone “smoothers,” presumably to work skins, have been found at Schöningen (*13*), Grotte des Contrebandiers (*14*), Pech-de-l’Azé I (*15*, *16*), Combe-Grenal (*17*), and Abri Peyrony (*16*). At the Grotte du Renne, several smoothers found in Châtelperronian layers associated with Neanderthals are dated to 45,000 to 42,000 cal B.P. (calibrated years before the present)—dates younger than 55,000 years ago are given here as cal B.P. (*18*)—and some of the smoothers bear ochre residues suggesting that they were used on ochred skin (*19*). Traditionally, archaeologists have associated the emergence of tailored clothes with the invention of eye needles made of bone (*20*, *21*). The initial appearance of eyed needles in the archaeological record of northern mid-latitude environments during the last glacial cycle is consistent with a primary function in providing thermal protection (*5*, *20*, *22*). The earliest known eyed needles appear ~40,000 cal B.P. in Siberia, ~38,000 cal B.P. in the Caucasus, ~30,000 cal B.P. in East Asia, and by 26,000 cal B.P. in Europe (*23*).

*Homo sapiens* arrived in Europe by ~45,000 cal B.P. (*24*, *25*). Before the dispersal of modern humans into Europe, the tools used by Neanderthals to prepare skins to cover themselves consisted of lithic hide-scrapers and rare borers, before the late appearance of bone tools in Châtelperronian contexts; skins may also have been treated with tanning agents (*26*). Therefore, the absence of bone awls and eyed needles cannot be argued as evidence for the absence of clothes altogether. Available evidence—including paleoenvironmental reconstructions, faunal remains, and ethnographic analogues—indicate that Neanderthals may have worn simple, poncho-style garments, although the presence of bone awls in Châtelperronian contexts could suggest a late adoption of fitted garments among some Neanderthal populations (*27*–*29*).

### Eyed needles

Production of eyed needles is a sophisticated, labor-intensive technological process for hunter-gatherers (*30*). If bone awls sufficed for sewing fitted garments and eyed needles were not necessarily invented to tailor clothes in the first instance, then the question arises as to what purpose(s) was sufficiently salient, or what needs were sufficiently pressing, to justify the extra investment. Some clues may be ascertained by considering the benefits of transforming an awl into an eyed needle.

1) In circumstances where finer sewing was desired or required, the process of inserting a thread by hand through a small hole became increasingly tedious, a challenge that was addressed by drilling an eye into the proximal end of the awl to carry the thread through.

2) As a consequence of the above technological innovation, eyed needles made sewing more efficient, by combining two separate processes into one: (i) the piercing of holes in hides and (ii) the threading of sinew or fiber through the holes.

The benefits of manufacturing eyed needles—facilitating finer sewing by hand and rendering the task of sewing more efficient—may pertain to adornment of clothes (*23*) and also the need for underwear in multilayered garment assemblages (*5*, *31*). These two different purposes actually coincide, since the thermal need for underwear corresponds to a more complete and continuous use of clothing, which, in turn, would favor a shift from decorating the skin surface to adorning the more visible surface of clothes (*5*). Eyed needles would also be useful in adding fur trim, which can serve both thermal and decorative purposes (*29*). Eyed needles were a technological advance that facilitated more effective thermal insulation and the transformation of clothing into dress. For these reasons, eyed needles were a tipping point in human prehistory.

## THERMAL ORIGINS

**Fig. 1.**
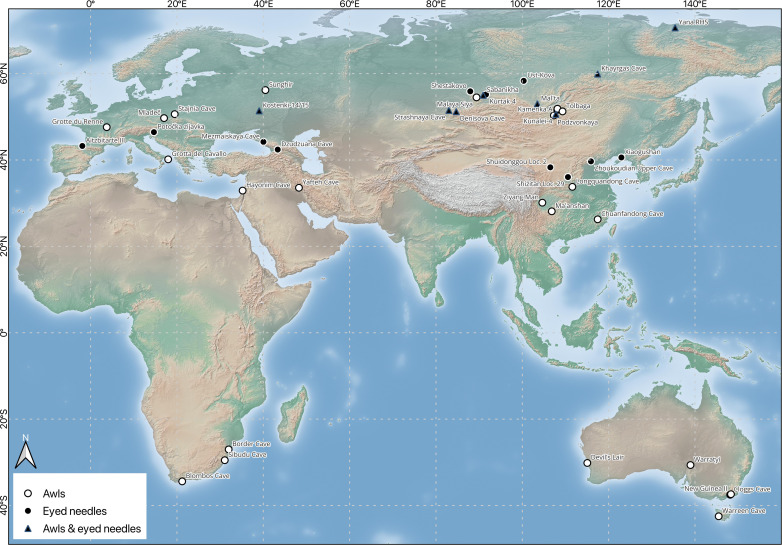
Locations for sites mentioned in the text with early bone awls and eyed needles. Map prepared by L. Doyon using QGIS v. 3.30.1-’s-Hertogenbosch (Free Software Foundation Inc., Boston; https://download.qgis.org/downloads/) with vector and raster from Natural Earth (https://www.naturalearthdata.com/).

A thermal basis for the development of clothing is consistent with evidence from a range of disciplines including ethnography [(*32*–*34*), text S1], archaeology (*5*, *31*, *35*), human physiology (*5*, *36*), clothing physiology (*5*, *37*), paleoclimatology (*38*–*40*), and entomology (*41*, *42*). Two forms of clothing—simple and complex—can be distinguished on the basis of thermal insulation properties (*5*). Simple clothes are structurally loose, offering limited protection from wind chill, whereas complex clothes are fitted and can also be multilayered. In terms of Paleolithic technologies, complex clothes are associated with dedicated hide-piercing implements, of which bone awls and eyed needles are well recognized. Bone awls appear in the archaeological record during the first half of the last glacial cycle in middle latitudes (Fig. 1), while the earliest eyed needles are restricted to the second half of the last glacial cycle (Table 1). In both cases, the geographical distributions reflect escalating physiological requirements for portable insulation as hominins occupied colder environments. Eyed needles accompanied the expansion of *H. sapiens* into Siberia (*43*, *44*) and subsequently across Beringia into the Americas (*35*, *45*).

**Table 1.** Sites with early eyed needles, including reported dates and latitude.

Site	Dating (cal B.P.)	Latitude	Region	Sources
Denisova Cave	40,000	51°N	Southern Siberia	Derevianko *et al.* (*113*); Kuzmin *et al.* (*100*)
Mezmaiskaya Cave	38,000	44°N	Caucasus	Golovanova *et al.* (*115*)
Zhoukoudian Upper Cave	35–33,000	40°N	Northeastern East Asia	Li *et al.* (*126*); d’Errico *et al.* (*50*)
Yana RHS	33,000	71°N	Northern Siberia	Pitulko and Pavlova (*44*); Shunkov *et al.* (*61*)
Xiaogushan	33–21,000	40°N	Northeastern East Asia	Huang *et al.* (*118*); Zhang *et al.* (*119*)
Shuidonggou 2	32,000	38°N	Northern central East Asia	Li *et al.* (*123*); Keates and Kuzmin (*124*)
Kunalei-4	32,000	51°N	Central Siberia	Fedorchenko and Belousova (*79*)
Sabanikha	31,000	56°N	Central Siberia	Shunkov (*61*); Fedorchenko and Belousova (*79*)
Potočka Cave	30,000	46°N	Central Europe	Odar (*127*); Anghelinu *et al.* (*128*)
Kostenki-15	30,000	51°N	Central Europe	Hoffecker (*35*)
Kurtak-4	29,000	55°N	Central Siberia	Shunkov *et al.* (*61*); Fedorchenko and Belousova (*79*)
Ust-Kova	28,000	58°N	Central Siberia	Fedorchenko and Belousova (*79*)
Aitzbitarte III	28–22,000	43°N	Western Europe	Calvo and Arrizabalaga (*129*)
Shestakovo	27,000	53°N	Central Siberia	Fedorchenko and Belousova (*79*)
Dzudzuana Cave	27–24,000	42°N	Caucasus	Bar-Yosef *et al.* (*116*)
Shizitan	26–23,000	36°N	Central eastern East Asia	Song *et al.* (*125*)
Khayrgas Cave	25,000	60°	Northeastern Siberia	Kuzmin *et al.* (*117*); Fedorchenko and Belousova (*79*)
Mal’ta	25,000	53°N	Southern Siberia	Shunkov *et al.* (*61*)
Strashnaya Cave	23,000	51°N	Southern Siberia	Shunkov *et al.* (*61*) Kuzmin *et al.* (*100*)
Broken Mammoth	14,000	64°N	Northwestern North America	Hoffecker (*45*); Hoffecker (*35*)

### Layers and underwear

The effectiveness of adding extra layers to improve insulation derives from the basic thermal principle of clothing, namely, trapping air near the skin surface to reduce the rate of convective heat loss. Using modern-day woven fabrics, and depending on the materials, adding one extra layer can almost double the insulation value (*36*, *37*). Holocene climates can be classified into clothing zones based on the number of layers required for people to safely endure outdoor winter conditions (*46*, *47*). Up to four layers are needed in the subarctic zone, for example, and four layers are mandatory for outdoor survival during Arctic winters when mean monthly temperatures are between −10°C and −20°C. However, these thresholds for extra layers are defined for garments made from woven materials, which provide less insulation, and are more prone to wind penetration, than the animal hides and furs used to manufacture Paleolithic garments. For this reason, the contemporary four-layer classification of clothing zones likely corresponds to a two-layer situation with Paleolithic garments (table S1).

## FROM COVERING TO DRESS

Regardless of whether or not a physiological incentive for underwear could be implicated in the advent of eyed needles, a reasonable presumption is that eyed needles reflect the emergence of a more general impetus for finer sewing. A thermal factor may be inferred from the correlation with exposure to colder environments, although this connection may not be limited to the manufacture of more thermally effective fitted clothing. As a consequence of the production of sophisticated clothing and more complete body covering, one repercussion of more regular and extensive body cover is that decoration of the human body for social purposes would necessarily be transferred from adorning the naked skin surface to decorating the surface of clothes (*5*).

A formative role for decoration and the adornment of clothing in the advent of eyed needles is supported by archaeological evidence (*4*, *48*–*50*). Specifically, the attachment of beads and other small decorative items to garments would favor the use of eyed needles. Acquisition of an adornment function meant that clothing underwent a transition from physical to social necessity—a transition to clothing as dress. This trend may be witnessed in the archaeological record as a greater range and diversity of beads, pendants, and other signs of adornment during the late Pleistocene in Eurasia, accompanying the development and morphological diversification of eyed needles (*23*, *50*–*54*).

## ARCHAEOLOGICAL EVIDENCE

The evolutionary context for eyed needles encompasses not only environmental parameters influencing the use of clothing but technological innovations preceding the advent of eyed needles and cultural factors affecting body adornment in the late Pleistocene. Evidence for body adornment is considered first, followed by technological precursors and alternatives for sewing, and archaeological evidence for the earliest eyed needles.

### Adornment

Adornment of the human body likely accompanied the emergence of *H. sapiens* between 300,000 and 200,000 years ago (*55*, *56*), and body adornment probably was present among other hominins, including Neanderthals. Evidence includes the use of ochre and the manufacture of decorative artifacts such as pierced marine shells and beads. Three broad phases in the evolution of body adornment can be inferred: adornment of the naked skin, wearing of decorative items (e.g., necklaces, earrings, and pendants), and adornment of clothes, with many technological steps involved—for instance, up to 10 steps from body painting to industrial bead production (*4*).

#### Ochre

The use of ochre for likely decorative purposes is documented in Africa from 100,000 years ago at Blombos Cave (*57*) and subsequently at other African sites (*58*) and in southwest Asia, at Qafzeh Cave 92,000 years ago (*59*). Three phases in the emergence of ochre use have been identified: sporadic use of ochre by hominins in Africa extending to at least 330,000 years ago, more frequent ochre use from 160,000 years ago, and more regular, habitual use from around 70,000 years ago (*60*). Use of ochre is documented at Kara-Bom, Central Altai (southern Siberia), ~43,000 cal B.P. (*61*) and in East Asia at Xiamabei ~40,000 cal B.P. (*62*), and also at the Zhoukoudian Upper Cave ~35,000 cal B.P. (*50*). Ochre was used from the first known human presence on the Australian continent, perhaps as early as 65,000 years ago (*63*). Among other hominins, use of ochre by Neanderthals is well documented (*64*, *65*).

#### Tattoos and cicatrices

Body adornment with tattoos and cicatrices is ethnographically widespread and, presumably, these practices extend deep into prehistoric times, although archaeological evidence is minimal. Among the oldest preserved tattoos are those found on the 5300-year-old Ötzi mummy in the southern Alps of Europe (*66*). Tattoos are preserved on mummies and depicted in artworks in many parts of the world from the mid-Holocene, from Africa to the Americas (*67*–*69*). The oldest tattooing tools include sharpened turkey bone tools from the Fernvale site in southeast North America, dated between 5500 and 1600 years ago (*70*). Tattooing tools are widespread throughout Polynesia, dating from 2700 years ago (*71*). Tiny quartz flakes that ethnographically were used to make cicatrices are reported from the Dauan site in the Torres Strait, dating to around 700 years ago (*72*).

#### Pierced shells and beads

**Fig. 2.**
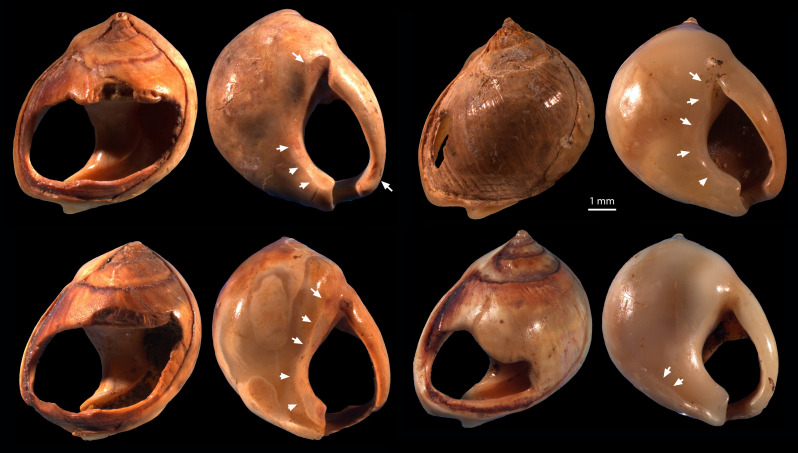
*Nassarius kraussianu* shell beads from Blombos Cave Still Bay layers, southern Africa, dated to approximately 73,000 to 70,000 years ago. Arrows indicate use-wear facets. Photos: F. d’Errico [modified after d’Errico *et al.* (*4*)].

Among the earliest known pieced ornaments are marine shell beads at Bizmoune and other northwest African sites, dating from the end of Marine Isotope Stage (MIS) 6 ~142,000 years ago and through MIS 5 (*49*, *73*, *74*). Shell beads have been recovered from southern African sites dating from ~90,000 years ago (*4*, *75*, *76*) (Fig. 2). Beads are also found in Châtelperronian contexts associated with Neanderthals (*77*). Early beads are dated to 43,000 cal B.P. at Bacho Kiro Cave in southeast Europe and Üçağızlı Cave in southwest Asia (*78*), and beads become common in Aurignacian assemblages from 42,000 cal B.P. (*52*). At the Yana RHS site within the Arctic Circle dated to 33,000 cal B.P., beads are found in association with bone awls, eyed needles, and needle cases, with some beads probably sewn onto clothes (*35*, *44*, *79*). Sewing of beads onto fitted garments is attested unequivocally at Sunghir in northwest Eurasia dating to 34,000 to 30,000 cal B.P., although the elaborate decorations were probably a special burial attire, not an everyday phenomenon (*80*, *81*). Use-wear evidence that beads may have been sewn onto clothes is reported at Üçağızlı Cave in southwest Asia 41,000 cal B.P. (*51*), and at Shuidonggou 2 in East Asia ~34,000 cal B.P. (*82*). At the Zhoukoudian Upper Cave, a pierced badger canine tooth has use-wear consistent with attachment to clothes (*67*). Shell beads occur in Island Southeast Asia (ISEA) from ~42,000 cal B.P. and become more common from the terminal Pleistocene (*83*, *84*). On the Australian continent, beads are documented from 30,000 cal B.P. (*85*), although Australian beads are likely unrelated to clothing, deployed typically in necklaces (*86*).

### Technological precursors

#### Awls

**Fig. 3.**
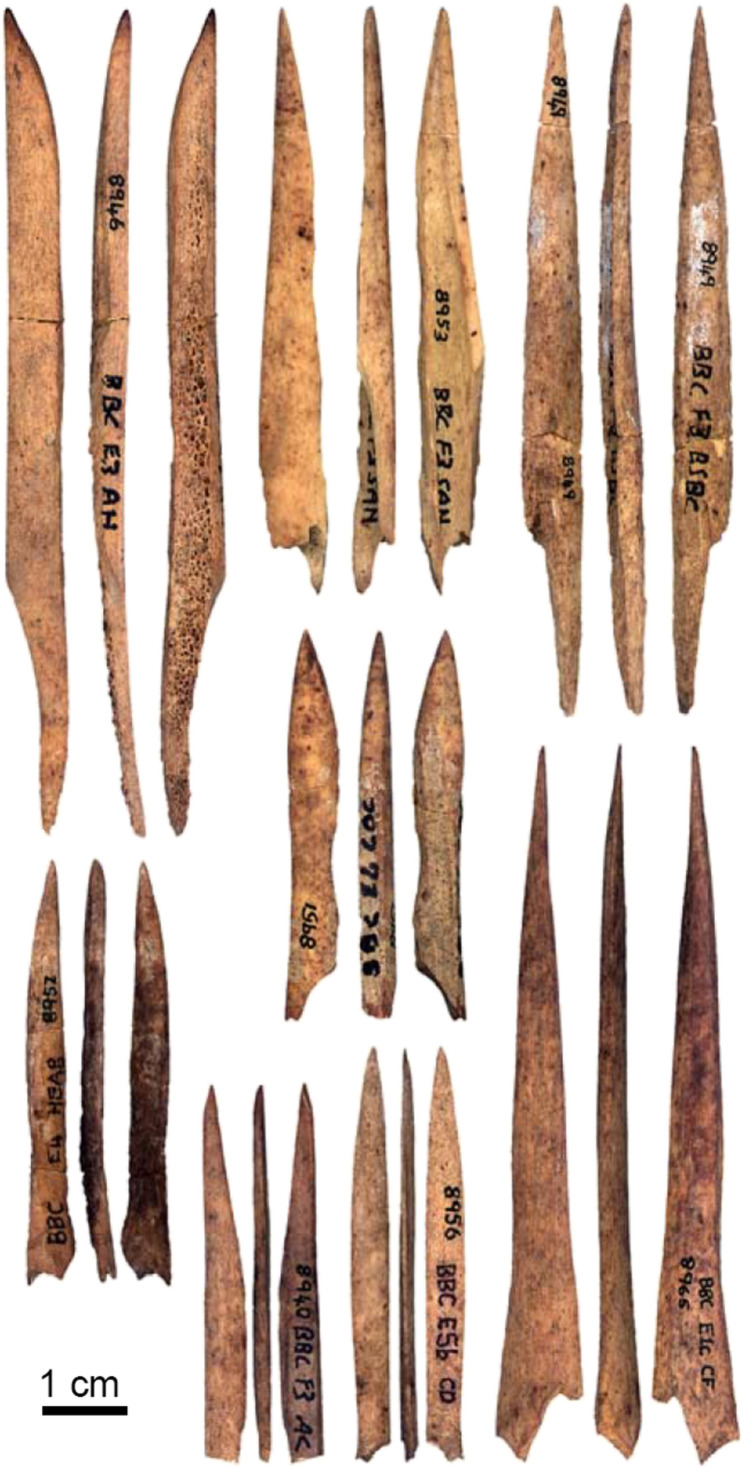
Bone awls from Blombos Cave, southern Africa, discovered in Still Bay layers dated to approximately 73,000 to 70,000 years ago. Photos: F. d’Errico.

Stone borers, which occur uncommonly in the Mousterian and become more frequent in the Upper Paleolithic, could have served in penetrating animal skins to make garments. However, the main technological precursor of the eyed needle is the bone awl. Technologically, eyed needles are modified awls, with a perforated hole (eye) to facilitate insertion of sinew or thread simultaneously with piecing a hole in a material for the purpose of sewing. Awls are essentially needles without eyes. Reflecting a likely role in sewing fitted clothes, the earliest awls in the archaeological record appear in concert with an increasing human presence in colder environments during the late Pleistocene. The earliest bone awls are found in southern Africa (Fig. 1), at Blombos Cave approximately 73,000 to 70,000 years ago (*87*) (Fig. 3), Sibudu Cave 61,000 years ago (*88*), and Border Cave 44,000 cal B.P. (*89*). At Blombos Cave, use-wear analyses suggest that 85% of bone tools functioned to “perforate fairly soft material such as well-worked hides, possibly during the manufacture of clothing and/or carrying bags” (*90*).

In Europe, early bone awls are found in Châtelperronian contexts approximately 45,000 to 42,000 cal B.P. at Grotte du Renne, likely produced by Neanderthals (*19*, *91*). Awls are dated to 42,000 cal B.P. at Stajnia Cave in central Europe (*92*), 40,000 cal B.P. at Grotta del Cavallo in southern Europe (*93*), and 31,000 cal B.P. at Mladeč, central Europe (*94*). In southwest Asia, awls are documented from 40,000 cal B.P. at Yafteh Cave (*95*) and 34,000 cal B.P. at Hayonim Cave (*96*). In northern Eurasia, early bone awls appear from 42,000 to 35,000 cal B.P. at Podzvonkaya, Kamenka A, Tolbaga, and Varvarina Gora in the Transbaikal region of southern Siberia (*61*, *79*, *97*, *98*), Kostenki-14 from 42,000 cal B.P. (*99*), Denisova Cave from at least 40,000 cal B.P. (*61*, *100*), Malaya Syia 36,000 cal B.P. (*61*), Sunghir 34,000 cal B.P. (*81*, *101*), Yana RHS 33,000 cal B.P. (*44*), Kunalei-4 32,000 cal B.P. (*79*), Sabanikha 31,000 cal B.P. (*79*), Mal’ta 25,000 cal B.P. (*61*), Khayrgas Cave 25,000 cal B.P. (*79*), and Strashnaya Cave 23,000 cal B.P. (*79*). Among the earliest awls in East Asia are those from Ma’anshan ~35,000 cal B.P., with polishing at the tips and microwear evidence of repeated grinding to maintain sharpness evident, consistent with piercing animal hides for clothing (*102*). A polished bone awl is reported from Longquan Cave dated to ~33,000 cal B.P. (*103*), and bone awls are also reported at Chuandong Cave between 40,000 and 30,000 cal B.P. (*104*) and at the Ziyang Man site ~30,000 cal B.P. (*105*). On the Australian continent, early bone awls are found in cooler southern regions, from between 40,000 and 38,000 cal B.P. at Warratyi (*106*), at Devil’s Lair in the southwest ~31,000 cal B.P. (*107*), in Tasmania at Warreen Cave ~31,000 cal B.P. (*108*), and in the southern highlands at Cloggs Cave ~23,000 cal B.P. (*109*) and nearby New Guinea II Cave ~21,000 cal B.P. (*110*).

#### Other sewing technologies

**Fig. 4.**
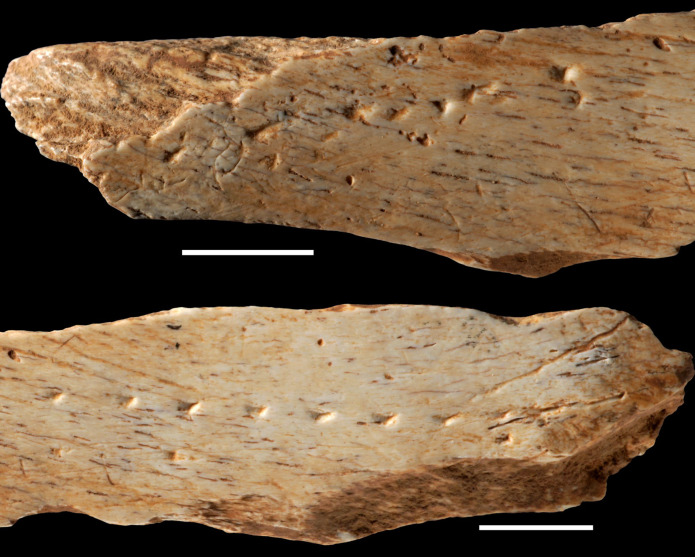
Puncture marks consistent with leather hole punching on a bone fragment at Canyars, Catalonia, dated to 39,600 cal B.P. Scale bars, 1 cm. Photos: L. Doyon, F. d’Errico.

A recent discovery from Canyars, an open-air site in southwest Europe, sheds light on the sewing technologies used in Europe before the introduction—or independent invention—of eyed needles (*111*). Microscopic analysis and experimental replication of aligned and grouped punctures on a large mammal hip bone fragment from this Aurignacian site dated to 39,600 cal B.P. (Fig. 4) indicate that the puncture marks were produced with different robust flint burins by indirect percussion with a hammer. The morphology, orientation, and arrangement of the punctures rule out the possibility that they were made to decorate the object or to record numerical information. The most parsimonious explanation for their presence on the surface of the bone is that they were made to perforate pieces of leather. The study therefore suggests that 14,000 years before the introduction of eye needles in Europe, Paleolithic hunter-gatherers were able to make tailored leather clothing and use it to cope with the harsh climatic conditions that affected Europe at that time. Another study has identified a specialized lithic tool, Noailles burins, from Gravettian contexts in Western Europe that probably served in tailoring clothes (*112*), adding further doubt about a necessary connection between eyed needles and tailoring garments.

### Early eyed needles

Mirroring the distribution of early bone awls, the world’s oldest eyed needles are found in colder regions during the late Pleistocene (Fig. 1), when average temperatures were declining as the global climate oscillated during late MIS 3, leading into the Last Glacial Maximum (LGM). The earliest eyed needles occur in northern Eurasia (Table 1), beginning from ~40,000 cal B.P. at Denisova Cave, which was occupied successively by Denisovans from 130,000 to 73,000 years ago and Neanderthals from >59,000 years ago to 50,000 cal B.P. (*35*, *79*, *100*, *113*). *H. sapiens* also occupied Denisova Cave, probably after 40,000 cal B.P., based on the occurrence of Initial Upper Paleolithic (IUP) lithic artifacts and genetic evidence, although uncertainties remain (*100*, *114*). Other sites with early eyed needles are Mezmaiskaya Cave in the Caucasus ~38,000 cal B.P. (*115*), Yana RHS in northern Siberia 33,000 cal B.P. (*43*, *44*, *79*), Kunalei-4 ~32,000 cal B.P. (*79*), Sabanikha 31,000 cal B.P. (*61*), Kostenki-15 30,000 cal B.P. (*35*, *45*), Kurtak-4 29,000 cal B.P. (*61*), Ust-Kova 28,000 cal B.P. (*79*), Shestakovo 27,000 cal B.P. (*79*), Dzudzuana Cave 27,000 to 24,000 cal B.P. (*116*), Khayrgas Cave ~25,000 cal B.P. (*79*, *117*), Mal’ta ~25,000 cal B.P. (*94*), and Strashnaya Cave by 23,000 cal B.P. (*61*, *100*).

In East Asia, eyed needles begin to appear at higher latitudes by ~33,000 cal B.P., for example, at Xiaogushan (*118*, *119*), following the arrival of *H. sapiens* in the region (*120*, *121*). *H. sapiens* was present as early as 45,000 cal B.P. at the site of Shiyu, where a perforated graphite disc may have functioned as a button on a cloak or a bag (*122*). A date of 32,000 cal B.P. is reported for a needle fragment from Shuidonggou 2 (*123*, *124*). Eyed needles are found also at Shizitan approximately 26,000 to 23,000 cal B.P. (*125*) and at Zhoukoudian Upper Cave, although dating of the latter is open to question (*48*, *118*, *126*). In Europe, early eyed needles occur at Potočka Cave 30,000 cal B.P. (*127*, *128*) and Aitzbitarte III between 28,000 and 22,000 cal B.P. (*129*). Eyed needles make an early appearance in the archaeological record of North America, dated to ~14,000 cal B.P. at the Broken Mammoth site in the northwest corner of the continent (*35*, *45*).

#### Morphological diversification

**Fig. 5.**
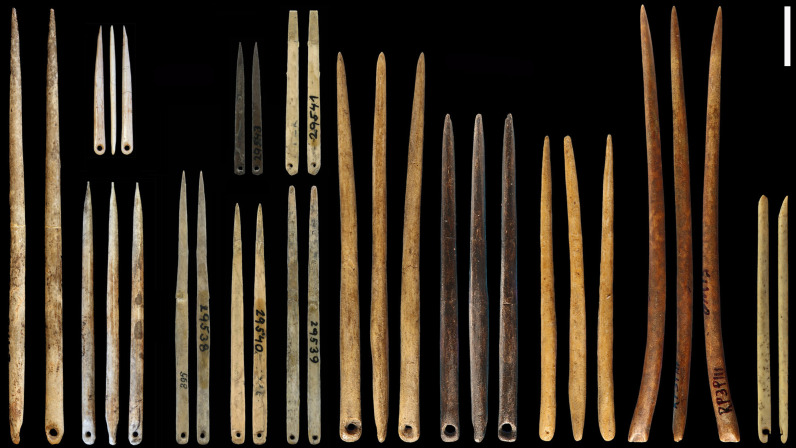
Morphological variation in the size and shape of Late Pleistocene eyed needles. Scale bar, 1 cm. Modified from d’Errico *et al.* (*23*).

A study of more than 200 late Pleistocene eyed needles from Eurasia and Paleoindian sites in North America identified a marked regional diversity in the size and shape of eyed needles and possible trends in morphology over time (Fig. 5) (*23*). The earliest eyed needles found in China are large and sturdy, suggesting that they were used for crafting thick, tailored clothing and robust leather objects. The size contrast between early needle specimens from Siberia and China suggests the possibility of independent invention in these regions, aligning with the different lithic technologies associated with the earliest eyed needles: the Early Upper Paleolithic in Siberia and Cores and Flakes Technology in China. Existing evidence indicates minimal variation in needle size throughout the Upper Paleolithic in Siberia. In contrast, smaller and flatter needles began to emerge in China between 26,000 and 23,000 cal B.P. The high latitude (71°N) site of Yana RHS in Siberia, dated to 33,000 cal B.P., has yielded a remarkable collection of Paleolithic sewing technologies, including 192 eyed needles and eyed needle fragments, 4 needle cases, and 79 bone awls and awl fragments. The eyed needles at Yana RHS manifest considerable morphological diversity. Eight varieties of eyed needles were identified, likely reflecting specialized functions in the production of complex clothing (e.g., underwear, mittens, and outer garments), shoes, and—in the case of the more robust needles with large eyes—sewing hides for constructing tents and covering habitation sites (*44*, *79*). The finer eyed needles would be useful in decorating garments, for instance, attaching pendants and beads, which were also found at the Yana RHS site.

The appearance of bone needles at a few Gravettian sites on the East European Plain dated to ~26,000 cal B.P. may represent either an independent invention or a geographic extension of early Siberian sewing traditions. No eyed needles have been found at numerous Gravettian sites in Western Europe (approximately 30,000 to 25,000 cal B.P.), despite substantial evidence of clothing and garment use during this period as demonstrated by numerous burials, personal ornaments, and evidence for the use of soft footwear, textiles, and nets (*130*–*132*). The relatively late appearance of eyed needles in Western Europe possibly reflects milder maritime climates compared to the more extreme seasonal ranges found in central Eurasia (*5*, *133*). Eyed needles appear in Western Europe with the Solutrean culture, corresponding with lower winter minimum temperatures in the LGM (*134*, *135*). During the subsequent Magdalenian period, there is a noticeable differentiation in size, with small, fine needles at one extreme and large, sturdy ones at the other, which suggests that the sewing kit of these Late Glacial hunter-gatherers contained needles of different caliper to perform specific tasks. Moreover, differences in the morphometric variation in needles found in Western Europe and in Central Europe suggest a strong pattern of regionalization in the manufacturing traditions. The morphological diversity across Eurasia may suggest independent origins in different regions, stylistic variations, cultural influences and drift, and, in some cases, demographic changes such as population replacement.

## PHYSICAL AND SOCIAL FUNCTIONS

Eyed needles make their appearance in the archaeological record of the Northern Hemisphere in a context of human exposure to increasingly colder conditions, from midway through the last glacial cycle. Eyed needles are currently unknown from the Southern Hemisphere during the Pleistocene, where thermal conditions were less extreme, even during the LGM. It is possible, however, that this absence of eyed needles could be explained by the poor preservation of organic remains in many localities of the Southern Hemisphere and/or by a historical imbalance in the intensity of research between the Northern Hemisphere contexts and the Global South. Nevertheless, an association between eyed needles and a physiological need for more thermally effective fitted clothing is apparent, although the nature of that association is not entirely clear from available evidence. A link with underwear has been posited (*5*), but despite the logic, convincing evidence for underwear in the late Pleistocene is scant. One corollary of more complete body covering with complex clothes is that the social functions of body decoration were necessarily shifted from the unclad skin surface onto the surface of clothes. This transition to clothing as dress was less likely to occur where clothing remained simple, as was the case in the higher latitudes of Australia (notably in Tasmania) and even in Tierra del Fuego (text S1). Acquisition of symbolic functions is not entirely excluded with simple clothes, but the traditional mode of symbolic expression was body decoration. Hence, simple clothing was less likely to persist beyond immediate thermal needs or solely for symbolic purposes. On the other hand, as an elaborate form of social display and communication, adornment of complex clothes may have allowed for increased regional diversification in cultural identities, with possible repercussions for social complexity (*4*). The thermal and social functions of eyed needles coincided, facilitating human colonization of high latitudes (including migration to the Americas across Beringia) and leading to the subsequent persistence of clothing in the Holocene for primarily social reasons.

### An evolutionary scenario

A thermal basis for clothing origins and clothing-related technological innovations is supported by evidence not only from archaeology but also from a range of disciplines including human physiology, paleoclimatology, ethnography, and genetic studies on clothing (body) lice. The coalescence of evidence from these independent sources constitutes a compelling argument for the thermal origin of clothing. A sequence of adaptive processes led from simple, utilitarian garments deployed on an ad hoc basis from the mid-Pleistocene to fitted garments in the last glacial cycle. Lithic borers and bone awls were the primary technology involved with fitted clothing, followed by eyed needles (perforated awls) as a refinement for finer sewing. Eyed needles underwent further morphological and, probably, functional diversification to become tools to perform both functional and symbolic practices.

**Fig. 6.**
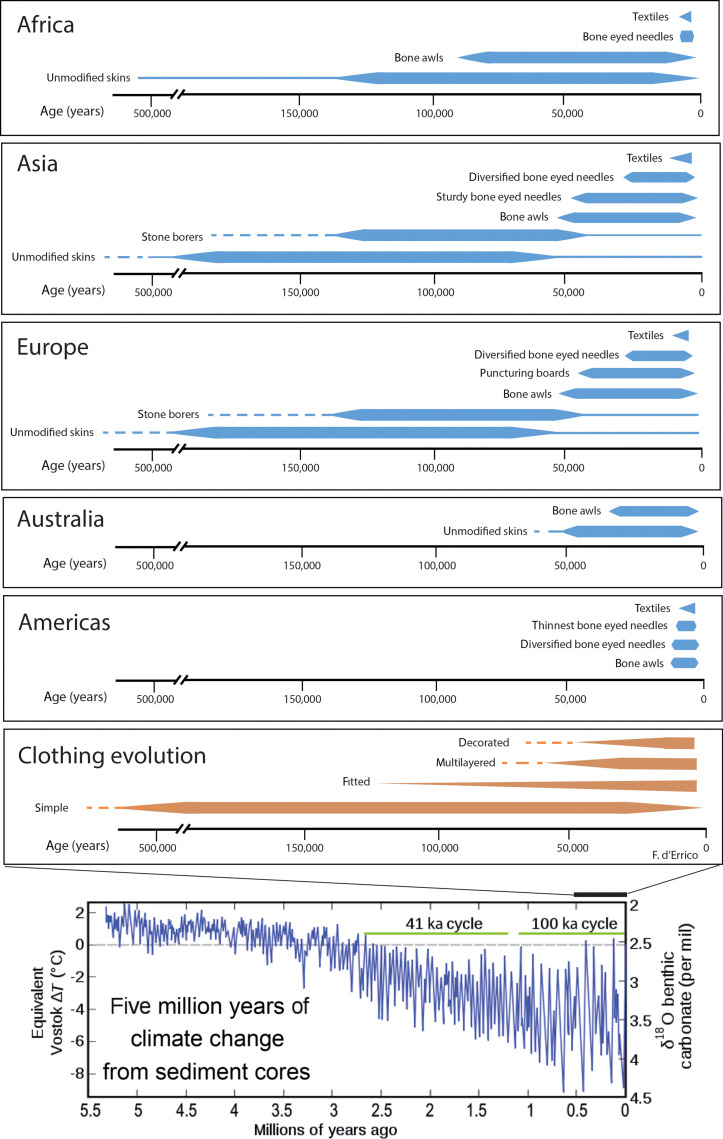
Major stages in the evolution of clothing. The main transitions in clothing, from ad hoc to routine use and from simple (loose) to complex (fitted ± layered) garments, together with the acquisition of decorative and symbolic functions. These transitions occurred within an environmental context of intensified global climate change from the mid-Pleistocene, associated with temperature fluctuations of increasing magnitude. Clothing charts by F. d’Errico; temperature chart from Brunetti and Prodi (*39*); reproduced under Creative Commons Attribution 4.0 License.

In the period that followed the first expansion of early humans out of Africa, the utilization of animal skins primarily served for protection against the elements. These skins were likely draped over the body quite loosely, providing basic insulation and protection. The use of simple, unfitted garments became more frequent due to climate change in the mid-Pleistocene, with the transition from 41,000-year to 100,000-year glacial cycles associated with temperature fluctuations of greater magnitude (Fig. 6). Archaeological evidence comprises lithic assemblages with increasing frequencies of hide-scraping tools for preparing skins, which otherwise were essentially unmodified, aside from any tanning or other treatments (*136*–*138*). The appearance of notched stone tools and borers indicate that human groups living in middle latitudes may have begun to develop basic sewing techniques and ways to wrap animal skins closely around the body 300,000 years ago, or earlier. With the invention of bone awls ~80,000 years ago, humans were able to create tailored and fitted garments by perforating skins with more precision. This advancement resulted in clothing that conforms better to the shape of the body and provides improved protection and comfort. The development in eastern Eurasia, ~40,000 cal B.P., of eyed needles allowed for more efficient sewing of clothes. This innovation enabled humans to create more complex stitched garments, enhancing both functionality and aesthetic appeal. Eyed needles were likely used not only for practical sewing but also for attaching decorative elements such as beads, shells, and feathers, adding cultural and aesthetic qualities to clothing.

## A SOCIAL CLIMATE FOR CLOTHING

Eyed needles are one of the most iconic of Paleolithic artifacts, traditionally regarded as confirming the development of fitted, or tailored, clothing in mid-latitude environments during the late Pleistocene. However, while the distribution and timing for the appearance of eyed needles in the archaeological record is consistent with an increased need for portable thermal insulation, recent discoveries and analyses demonstrate that production of fitted garments does not require eyed needles. Instead, a more nuanced perspective is required to account for the advent of eyed needles.

Fitted garments were likely manufactured with awls, burins, and other devices before the advent of eyed needles. As a technological innovation that facilitates more efficient and finer sewing, the manufacture of eyed needles indicates an intensified demand for intricate sewing. In an environmental context of heightened clothing requirements, two functions that entail intricate sewing may have favored eyed needles. One is underwear, providing additional thermal insulation in multilayered garment assemblages. Another function for eyed needles relates to adornment of clothes by the attachment of beads and other ornaments onto the surface of garments. This latter function reflects the emergence of clothing as dress, a logical consequence of the more complete body covering required for physiological reasons in mid-latitude Eurasia toward and during the LGM. The two functions are not mutually exclusive, and both coincide with the increased body covering associated with the relatively late advent of complex clothing in hominin evolution.

The importance of eyed needles lies not in tailoring clothes but rather a further elaboration of clothing that, while technologically a small step, was to prove a quantum leap in human societies where clothing was used on a regular basis. Together with the largely invisible development of underwear and the more obscure genesis of modesty as a motive for covering the human body regardless of climate, the transition to clothing as dress transformed clothing from a physical to a social necessity, ensuring the continued use of clothing up to the present.

## Supplementary Materials

### This PDF file includes:

Text S1 Table S1 References
