# Supplementary material for: Paleolithic eyed needles and the evolution of dress
Source: Sci Adv. 2024 Jun 28;10(26):eadp2887. doi: 10.1126/sciadv.adp2887 (PMC11212769; doi:10.1126/sciadv.adp2887)
Supplement: Supplementary file 1 — Text S1 Table S1 References [file sciadv.adp2887_sm.pdf]

Science Advances
AAAS

Supplementary Materials for

Paleolithic eyed needles and the evolution of dress

Ian Gilligan *et al.*

Corresponding author: Ian Gilligan, ian.gilligan@sydney.edu.au

Sci. Adv. **10**, eadp2887 (2024)
DOI: 10.1126/sciadv.adp2887

This PDF file includes:

Text S1
Table S1
References

Supplementary Materials

Text S1

Ethnographic insights

The use of ethnography in prehistoric archaeology has a long and controversial history. Nevertheless, a general consensus has emerged which stipulates that providing ethnographic data are not misused as an analogy or template for reconstructing prehistory, evidence from recent non-industrialized societies can offer useful insights and assist in generating and testing hypotheses (*139–141*).

Inuit clothing

The sophisticated clothing of the Inuit serves to illustrate the need for carefully tailored, finely-sewn garments in Arctic environments (*30*, *142*). The Inuit employed both awls and eyed needles, using awls on tougher hides (e.g., sealskins) and to initiate holes in softer skins (e.g., caribou hides). Awls were made from the bones of caribou, seal, and bear, and from the tusks of narwhal or walrus; larger awls were often decorated with intricate markings and engravings. Eyed needles were reserved for the final sewing of precisely cut hide pieces, and these fragile tools were stored in needle cases, typically decorated with elaborate cultural motifs. Thimbles were used in sewing, made from the tough skin of seal or walrus, or from a caribou toe bone, or musk-ox horn. Decoration of traditional Inuit clothing was achieved mainly by combining hides and furs of differing colours in varied patterns; less commonly, garments were adorned with sewn beads, bird beaks, or feathers. Durability was an issue: a

set of garments would usually last for only one year, with two different sets required, one each for winter (generally two layers) and summer. The manufacture and maintenance of these complex clothing assemblages was an ongoing daily task. In hunter-gatherer contexts where such complex clothing is mandatory for survival, any technological innovation—eyed needles, for instance—that increases the production efficiency of manufacturing fitted clothing is valuable, and the evident utility will justify the extra effort expended in its manufacture.

Ainu clothing

The Ainu of northern Japan typically wore two layers of clothing, with outer garments and underwear made from a wide range of mammal, fish and bird skins, as well as from woven plant fibres (e.g., nettle) and bark cloth (from the inner bark of the Japanese elm). Eyed needles were stored in decorated needle cases, and adornment of outer garments was exquisite. In addition to combining different colours in sewn skin garments, embroidery and appliqué techniques were applied to cloth materials. Beads were used mainly in necklaces and earrings. Tattooing of exposed skin areas (mainly hands, wrists, forearm, face and lips) was widespread, especially among females, using delicate obsidian-blade knives (*143*).

Tierra del Fuego

Simpler forms of clothing were employed in Tierra del Fuego on the southernmost edge of South America, where single-layered capes and cloaks—sometimes sewn from multiple skins—sufficed as portable insulation among the Ona and Yahgan peoples (*144*, *145*). Notably, eyed needles were absent, with the Yahgan using awls for sewing (*144*). In all likelihood, the Fuegians benefitted from enhanced biological adaptations to cold that had developed over millennia. To the astonishment of Charles Darwin when he visited Tierra del Fuego on the *Beagle* in 1832, the Fuegians often went unclad (*146*). Clothes functioned as

protection from colder temperatures and wind chill, while dressing was achieved mainly with body painting. In order to get properly dressed on ceremonial occasions, the Fuegians would remove any clothes and paint themselves (*145*).

Australian evidence

Eyed needles were absent on the Australian continent, where the Indigenous peoples usually wore no clothes routinely and adorned themselves with body painting, tattooing, scarification, beads, necklaces, and nose bones, among other decorative techniques. Clothing was limited to the cooler southern regions, employed on a pragmatic basis when needed for warmth (*147*, *148*). Garments were single-layered, worn loosely over the shoulders, made typically from a kangaroo or wallaby skin or, in the southern highlands, possum-fur skins sewn together with awls to make large cloaks; these skins served otherwise as rugs and blankets. Archaeological evidence indicates the earliest bone awls in Australia first appeared in southern areas as temperatures declined towards the LGM (*106-110*, *147*). Notably, thermal conditions during the late Pleistocene in Australia were milder than in mid-latitude Eurasia. Also relevant is evidence in Tasmania, which was joined to the southern margin of the continent when sea levels were lower during the late Pleistocene. Bone awls are documented in Tasmania from 31,000 cal BP, likely deployed in the sewing together of small wallaby skins to make larger cloaks. Intriguingly, bone awls (along with small stone hide-scrapers, known as thumbnail scrapers, which dominate Tasmanian lithic assemblages in the late Pleistocene) disappear from the Tasmanian archaeological record in the early-mid Holocene, as the climate ameliorated (*108*, *149*). Paralleling the situation in Tierra del Fuego (and, likewise, to the surprise of the first European visitors), the Tasmanians were routinely unclad during the late Holocene (*147*, *150*). Like the Fuegians, the Indigenous Tasmanians probably had developed biological cold adaptations over 35,000 years in the region, allowing them to manage comfortably without the regular use of clothing (*147*). Collectively, the evidence from

Australia is significant for several reasons: the presence of bone awls but not eyed needles, the continuing decoration of the unclad human body rather than using clothes as dress, the absence of fitted garments, and the failure of clothing to acquire social and cultural functions that elsewhere—particularly in the Northern Hemisphere—led to a decoupling of clothing from climate. In Australia, clothing remained coupled to climate and did not become dress.

Table S1

Contemporary clothing layer zones.

Contemporary global climates are divided into four zones for minimum clothing layer requirements. Shown also are approximate winter mean monthly temperatures and latitudes, the suggested clothing layer equivalents for non-woven materials (animal hides and furs) during the Late Pleistocene, clothing forms, and associated osseous technologies; based on Gilligan (2010) and Eicher and Evenson (2015) (*5*,*47*).

Clothing zone (layers)	Climate zone	Winter temp. (°C)	Latitude (°N)	Pleistocene clothing layers	Clothing form	Bone technology
0	tropics	> 20	0 to 20	0	none	nil
1	sub-tropics	20 to 10	20 to 30	0 to 1	none<br>simple	nil<br>nil
2	cool temperate	10 to 0	30 to 40	1	simple<br>complex/ fitted	nil<br>awl
3	cold temperate	0 to -10	40 to 50	1 to 2	complex/ fitted<br>complex/ layered	awl<br>eyed needle
4	subarctic arctic	-10 to -20	50 +	2	complex/ fitted<br>complex/ layered	awl<br>eyed needle

REFERENCES AND NOTES

1. R. A. Joyce, Archaeology of the body. *Annu. Rev. Anthropol.* **34**, 139–158 (2005).

2. K. Laland, B. Matthews, M. W. Feldman, An introduction to niche construction theory. *Evol. Ecol.* **30**, 191–202 (2016).

3. A. Nowell, A. Cooke, in *The Oxford Handbook of Human Symbolic Evolution*, N. Gontier, A. Lock, C. Sinha, Eds. (Oxford Univ. Press, 2021), pp. 400–428.

4. F. d'Errico, K. L. van Niekerk, L. Geis, C. S. Henshilwood, New Blombos Cave evidence supports a multistep evolutionary scenario for the culturalization of the human body. *J. Hum. Evol.* **184**, 103438 (2023).

5. I. Gilligan, The prehistoric development of clothing: Archaeological implications of a thermal model. *J. Archaeol. Method Theory* **17**, 15–80 (2010).

6. M. L. S. Sørensen, Reading dress: The construction of social categories and identities in Bronze Age Europe. *Eur. J. Archaeol.* **5**, 93–114 (1997).

7. C. L. White, M. C. Beaudry, in *International Handbook of Historical Archaeology*, D. Gaimster, T. Majewski, Eds. (Springer, 2009), pp. 209–225.

8. I. Gilligan, Clothing and hypothermia as limitations for midlatitude hominin settlement during the Pleistocene: A comment on Hosfield 2016. *Curr. Anthropol.* **58**, 534–535 (2017).

9. L. H. Keeley, *Experimental Determination of Stone Tool Uses: A Microwear Analysis* (University of Chicago Press, 1980).

10. C. Lemorini, F. Venditti, E. Assaf, Y. Parush, R. Barkai, A. Gopher, The function of recycled lithic items at late Lower Paleolithic Qesem Cave, Israel: An overview of the use-wear data. *Quat. Int.* **361**, 103–112 (2015).

11. V. Rots, B. L. Hardy, J. Serangeli, N. J. Conard, Residue and microwear analyses of the stone artifacts from Schöningen. *J. Hum. Evol.* **89**, 298–308 (2015).

12. I. Verheijen, B. M. Starkovich, J. Serangeli, T. van Kolfschoten, N. J. Conard, Early evidence for bear exploitation during MIS 9 from the site of Schöningen 12 (Germany). *J. Hum. Evol.* **177**, 103294 (2023).

13. M.-A. Julien, B. Hardy, M. C. Stahlschmidt, B. Urban, J. Serangeli, N. J. Conard, Characterizing the Lower Paleolithic bone industry from Schöningen 12 II: A multi-proxy study. *J. Hum. Evol.* **89**, 264–286 (2015).

14. E. Y. Hallett, C. W. Marean, T. E. Steele, E. Álvarez-Fernández, Z. Jacobs, J. N. Cerasoni, V. Aldeias, E. M. L. Scerri, D. I. Olszewski, M. A. El Hajraoui, H. L. Dibble, A worked bone assemblage from 120,000–90,000 year old deposits at Contrebandiers Cave, Atlantic Coast, Morocco. *iScience* **24**, 102988 (2021).

15. M. Soressi, S. P. McPherron, M. Lenoir, T. Dogandžić, P. Goldberg, Z. Jacobs, Y. Maigrot, N. L. Martisius, C. E. Miller, W. Rendu, M. Richards, M. M. Skinner, T. E. Steele, S. Talamo, J.-P. Texier, Neandertals made the first specialized bone tools in Europe. *Proc. Natl. Acad. Sci. U.S.A.* **110**, 14186–14190 (2013).

16. N. L. Martisius, F. Welker, T. Dogandžić, M. N. Grote, W. Rendu, V. Sinet-Mathiot, A. Wilcke, S. J. P. McPherron, M. Soressi, T. E. Steele, Non-destructive ZooMS identification reveals strategic bone tool raw material selection by Neandertals. *Sci. Rep.* **10**, 7746 (2020).

17. E. Tartar, A. Legrand-Pineau, E. Claud, S. Costamagno, E. Discamps, J.-P. Faivre, Informal but specialized: Mousterian bone hideworking tools from Combe-Grenal (Dordogne, France). *PaleoAnthropology* **2022**, 211–236 (2022).

18. P. J. Reimer, W. E. N. Austin, E. Bard, A. Bayliss, P. G. Blackwell, C. Bronk Ramsey, M. Butzin, H. Cheng, R.L. Edwards, M. Friedrich, P. M. Grootes, T. P. Guilderson, I. Hajdas, T. J. Heaton, A. G. Hogg, K. A. Hughen, B. Kromer, S. W. Manning, R. Muscheler, J. G. Palmer, C. Pearson, R. W. Reimer, D. A. Richards, E. M. Scott, J. R. Southon, C. S. M. Turney, L. Wacker, F. Adolphi, U. Büntgen, M. Capano, S.M. Fahrni, A. Fogtmann-Schulz, R. Friedrich, P. Köhler, S. Kudsk, F. Miyake, J. Olsen, F. Reinig, M. Sakamoto, A. Sookdeo, S. Talamo, The IntCal20 Northern Hemisphere radiocarbon age calibration curve (0–55 cal kbp). *Radiocarbon* **62**, 725–757 (2020).

19. M. Julien, M. Vanhaeren, F. d'Errico, in *Le Châtelperronien de La Grotte Du Renne (Arcy-Sur-Cure, Yonne, France)*, M. Julien, F. David, M. Girard, A. Roblin-Jouve, Eds. (Supplément à Paléo, 2019), pp. 139–190.

20. D. de Sonneville–Bordes, in *France Before the Romans*, S. Piggot, G. Daniel, C. McBurney, Eds. (Thames and Hudson, 1973), pp. 30–60.

21. D. Stordeur-Yedid, *Les Aiguilles à Chas au Paléolithique* (CNRS, 1979).

22. S. A. Semenov, *Prehistoric Technology: An Experimental Study of the Oldest Tools and Artefacts from Traces of Manufacture and Wear* (Cory, Adams and MacKay, 1964).

23. F. d'Errico, L. Doyon, S. Zhang, M. Baumann, M. Lázničová-Galetová, X. Gao, F. Chen, Y. Zhang, The origin and evolution of sewing technologies in Eurasia and North America. *J. Hum. Evol.* **125**, 71–86 (2018).

24. J.-J. Hublin, N. Sirakov, V. Aldeias, S. Bailey, E. Bard, V. Delvigne, E. Endarova, Y. Fagault, H. Fewlass, M. Hajdinjak, B. Kromer, I. Krumov, J. Marreiros, N. L. Martisius, L. Paskulin, V. Sinet-Mathiot, M. Meyer, S. Pääbo, V. Popov, Z. Rezek, S. Sirakova, M. M. Skinner, G. M. Smith, R. Spasov, S. Talamo, T. Tuna, L. Wacker, F. Welker, A. Wilcke, N. Zahariev, S. P. McPherron, T. Tsanova, Initial Upper Palaeolithic *Homo sapiens* from Bacho Kiro Cave, Bulgaria. *Nature* **581**, 299–302 (2020).

25. D. Mylopotamitaki, M. Weiss, H. Fewlass, E. I. Zavala, H. Rougier, A. P. Sümer, M. Hajdinjak, G. M. Smith, K. Ruebens, V. Sinet-Mathiot, S. Pederzani, E. Essel, F. S. Harking, H. Xia, J. Hansen, A. Kirchner, T. Lauer, M. Stahlschmidt, M. Hein, S. Talamo, L. Wacker, H. Meller, H. Dietl, J. Orschiedt, J.V. Olsen, H. Zeberg, K. Prüfer, J. Krause, M. Meyer, F. Welker, S.P. McPherron, T. Schüler, J.-J. Hublin. *Homo sapiens* reached the higher latitudes of Europe by 45,000 years ago. *Nature* **626**, 341–346 (2024).

26. H. Meller, in *Erkenntnisjäger: Kultur und Umwelt des Frühen Menschen,* J. M. Burdukiewicz, L Fiedler, W.-D. Heinrich, A. Justus, E. Brühl, Eds. (Landesmuseum fur Vorgeschichte, 2003), pp. 697–702.

27. I. Gilligan, Neanderthal extinction and modern human behaviour: The role of climate change and clothing. *World Archaeol.* **39**, 499–514 (2007).

28. N. Wales, Modeling Neanderthal clothing using ethnographic analogues. *J. Hum. Evol.* **63**, 781–795 (2012).

29. M. Collard, L. Tarle, D. Sandgathe, A. Allan, Faunal evidence for a difference in clothing use between Neanderthals and early modern humans in Europe. *J. Anthropol. Archaeol.* **44**, 235–246 (2016).

30. B. K. Issenman, *Sinews of Survival: The Living Legacy of Inuit Clothing* (University of British Columbia Press, 1997).

31. I. Gilligan, *Climate, Clothing, and Agriculture in Prehistory: Linking Evidence, Causes, and Effects* (Cambridge Univ. Press, 2019).

32. E. H. Man, *On the Aboriginal Inhabitants of the Andaman Islands* (Royal Anthropological Institute, 1932).

33. R. B. Lee, *The !Kung San: Men, Women, and Work in a Foraging Society* (Cambridge Univ. Press, 1980).

34. T. Nomokonova, R. J. Losey, A. V. Gusev, G. Kohut, S. Razdymakha, L. Vozelova, A. V. Plekhanov, The one-eyed Elder woman stitches an ornament: Needles, needle cases, and women from the Iamal-Nenets region of Arctic Siberia. *J. Anthropol. Archaeol.* **74**, 101589 (2024).

35. J. F. Hoffecker, *Modern Humans: Their African Origin and Global Dispersal* (Columbia Univ. Press, 2017).

36. K. C. Parsons, *Human Thermal Environments: The Effects of Hot, Moderate, and Cold Environments on Human Health, Comfort and Performance* (Taylor & Francis, 2014).

37. L. H. Newburgh, Ed. *Physiology of Heat Regulation and the Science of Clothing* (W. B. Saunders, 1949).

38. I. K. Seierstad, P. M. Abbott, M. Bigler, T. Blunier, A. J. Bourne, E. Brook, S. L. Buchardt, C. Buizert, H. B. Clausen, E. Cook, D. Dahl-Jensen, S. M. Davies, M. Guillevic, S. J. Johnsen, D. S. Pedersen, T. P. Popp, S. O. Rasmussen, J. P. Severinghaus, A. Svensson, B. M. Vinther, Consistently dated records from the Greenland GRIP, GISP2 and NGRIP ice cores for the past 104 ka reveal regional millennial-scale $\delta^{18}O$ gradients with possible Heinrich event imprint. *Quat. Sci. Rev.* **106**, 29–46 (2014).

39. M. Brunetti, F. Prodi, The climate system. *EPJ Web Conf.* **98**, 02001 (2015).

40. J. D. Annan, J. C. Hargreaves, T. Mauritsen, A new global surface temperature reconstruction for the Last Glacial Maximum. *Clim. Past* **18**, 1883–1896 (2022).

41. R. Kittler, M. Kayser, M. Stoneking, Molecular evolution of *Pediculus humanus* and the origin of clothing. *Curr. Biol.* **13**, 1414–1417 (2003).

42. M. A. Toups, A. Kitchen, J. E. Light, D. L. Reed, Origin of clothing lice indicates early clothing use by anatomically modern humans in Africa. *Mol. Biol. Evol.* **28**, 29–32 (2011).

43. Y. V. Kuzmin, Siberia at the Last Glacial Maximum: Environment and archaeology. *J. Archaeol. Res.* **16**, 163–221 (2008).

44. V. V. Pitulko, E. Y. Pavlova, Upper Palaeolithic sewing kit from the Yana site, Arctic Siberia. *Strat. Plus* **2019**, 157–224 (2019).

45. J. F. Hoffecker, *A Prehistory of the North: Human Settlement of the Higher Latitudes* (Rutgers Univ. Press, 2005).

46. B. W. Olesen, in *Environmental Ergonomics: The Ergonomics of Human Comfort, Health and Performance in the Thermal Environment*, Y. Tochihara , T. Ohnaka, Eds. (Elsevier, 2005), pp. 479–506.

47. J. B. Eicher, S. L. Evenson, *The Visible Self: Global Perspectives on Dress, Culture, and Society* (Fairchild, 2015).

48. A. Bouzouggar, N. Barton, M. Vanhaeren, F. d'Errico, S. Collcutt, T. Higham, E. Hodge, S. Parfitt, E. Rhodes, J.-L. Schwenninger, C. Stringer, E. Turner, S. Ward, A. Moutmir, A. Stambouli, 82,000-year-old shell beads from north Africa and implications for the origins of modern human behavior. *Proc. Natl. Acad. Sci. U.S.A.* **104**, 9964–9969 (2007).

49. F. d'Errico, M. Vanhaeren, N. Barton, A. Bouzouggar, H. Mienis, D. Richter, J.-J. Hublin, S. P. McPherron, P. Lozouet, Additional evidence on the use of personal ornaments in the middle Paleolithic of north Africa. *Proc. Natl. Acad. Sci. U.S.A.* **106**, 16051–16056 (2009).

50. F. d'Errico, A. P. Martí, Y. Wei, X. Gao, M. Vanhaeren, L. Doyon, Zhoukoudian Upper Cave personal ornaments and ochre: Rediscovery and reevaluation. *J. Hum. Evol.* **161**, 103088 (2021).

51. M. C. Stiner, S. L. Kuhn, E. Güleç, Early Upper Paleolithic shell beads at Üçağızlı Cave I (Turkey): Technology and the socioeconomic context of ornament life-histories. *J. Hum. Evol.* **64**, 380–398 (2013).

52. M. Vanhaeren, F. d'Errico, Aurignacian ethno-linguistic geography of Europe revealed by personal ornaments. *J. Archaeol. Sci.* **33**, 1105–1128 (2006).

53. L. Lbova, Personal ornaments as markers of social behavior, technological development and cultural phenomena in the Siberian early upper Paleolithic. *Quat. Int.* **573**, 4–13 (2021).

54. J. Baker, S. Rigaud, D. Pereira, L.A. Courtenay, F. D'Errico, Evidence from personal ornaments suggest nine distinct cultural groups between 34,000 and 24,000 years ago in Europe. *Nat. Hum. Behav.* **8**, 431–444 (2024).

55. C. M. Schlebusch, H. Malmström, T. Günther, P. Sjödin, A. Coutinho, H. Edlund, A. R. Munters, M. Vicente, M. Steyn, H. Soodyall, M. Lombard, M. Jakobsson, Southern African ancient genomes estimate modern human divergence to 350,000 to 260,000 years ago. *Science* **358**, 652–655 (2017).

56. C. M. Vidal, C. S. Lane, A. Asrat, D. N. Barfod, D. F. Mark, E. L. Tomlinson, A. Z. Tadesse, G. Yirgu, A. Deino, W. Hutchinson, A. Mounier, C. Oppenheimer, Age of the oldest known *Homo sapiens* from eastern Africa. *Nature* **601**, 579–583 (2022).

57. C. S. Henshilwood, F. d’Errico, K. L. van Niekerk, Y. Coquinot, Z. Jacobs, S.-E. Lauritzen, M. Menu, R. García-Moreno, A 100,000-year-old ochre-processing workshop at Blombos Cave, South Africa. *Science* **334**, 219–222 (2011).

58. F. d’Errico, R. G. Moreno, R. F. Rifkin, Technological, elemental and colorimetric analysis of an engraved ochre fragment from the Middle Stone Age levels of Klasies River Cave 1, South Africa. *J. Archaeol. Sci.* **39**, 942–952 (2012).

59. E. Hovers, S. Ilani, O. Bar-Yosef, B. Vandermeersch, An early case of color symbolism. *Curr. Anthropol.* **44**, 491–522 (2003).

60. R. Dapschauskas, M. B. Göden, C. Sommer, A. W. Kandel, The emergence of habitual ochre use in Africa and its significance for the development of ritual behavior during the Middle Stone Age. *J. World Prehist.* **35**, 233–319 (2022).

61. M. V. Shunkov, A. Y. Fedorchenko, M. B. Kozlikin, A. P. Derevianko, Initial Upper Palaeolithic ornaments and formal bone tools from the East Chamber of Denisova Cave in the Russian Altai. *Quat. Int.* **559**, 47–67 (2020).

62. F.-G. Wang, S.-X. Yang, J.-Y. Ge, A. Ollé, K.-L. Zhao, J.-P. Yue, D. E. Rosso, K. Douka, Y. Guan, W.-Y. Li, H.-Y. Yang, L.-Q. Liu, F. Xie, Z.-T. Guo, R.-X. Zhu, C.-L. Deng, F. d’Errico, M. Petraglia, Innovative ochre processing and tool use in China 40,000 years ago. *Nature* **603**, 284–289 (2022).

63. C. Clarkson, Z. Jacobs, B. Marwick, R. Fullagar, L. Wallis, M. Smith, R. G. Roberts, E. Hayes, K. Lowe, X. Carah, S. A. Florin, J. McNeil, D. Cox, L. J. Arnold, Q. Hua, J. Huntley, H. E. A. Brand, T. Manne, A. Fairbairn, J. Shulmeister, L. Lyle, M. Salinas, M. Page, K. Connell, G. Park, K. Norman, T. Murphy, C. Pardoe, Human occupation of northern Australia by 65,000 years ago. *Nature* **547**, 306–310 (2017).

64. M. Soressi, F. d’Errico, in *Les Neandertaliens: Biologie et Cultures*, B. Vandermeersch, B. Maureille, Eds. (CTHS, 2007), pp. 297–309.

65. W. Roebroeks, M. J. Sier, T. K. Nielsen, D. De Loecker, J. M. Parés, C. E. S. Arps, H. J. Mücher, Use of red ochre by early Neandertals. *Proc. Natl. Acad. Sci. U.S.A.* **109**, 1889–1894 (2012).

66. A. Deter-Wolf, B. Robitaille, L. Krutak, S. Galliot, The world's oldest tattoos. *J. Archaeol. Sci. Rep.* **5**, 19–24 (2016).

67. N. V. Polosmak, Tattoos in the Pazyryk world. *Archaeol. Ethnol. Anthropol. Eurasia* **4**, 95–102 (2000).

68. G. J. Tassie, Identifying the practice of tattooing in ancient Egypt and Nubia. *Pap. Inst. Archaeol.* **14**, 85–101 (2003).

69. L. Krutak, *Tattoo Traditions of Native North America: Ancient and Contemporary Expressions of Identity* (LM Publishers, 2014).

70. A. Deter-Wolf, T. M. Peres, S. Karacic, Ancient Native American bone tattooing tools and pigments: Evidence from central Tennessee. *J. Archaeol. Sci. Rep.* **37**, 103002 (2021).

71. G. Clark, M. C. Langley, Ancient tattooing in Polynesia. *J. Isl. Coast. Archaeol.* **15**, 407–420 (2020).

72. I. J. McNiven, Dauan 4 and the emergence of ethnographically-known social arrangements across Torres Strait during the last 600-800 years. *Aust. Archaeol.* **62**, 1–12 (2006).

73. M. Vanhaeren, F. d'Errico, C. Stringer, S. L. James, J. A. Todd, H. K. Mienis, Middle Paleolithic shell beads in Israel and Algeria. *Science* **312**, 1785–1788 (2006).

74. E. M. Sehasseh, P. Fernandez, S. Kuhn, M. Stiner, S. Mentzer, D. Colarossi, A. Clark, F. Lanoe, M. Pailes, D. Hoffmann, A. Benson, E. Rhodes, M. Benmansour, A. Laissaqui, I. Ziani, P. Vidal-Matutano, J. Morales, Y. Djellal, B. Longet, J.-J. Hublin, M. Mouhiddine, F.-Z. Rafi, K. B. Worthey, I. Sanchez-Morales, N. Ghayati, A. Bouzouggar, Early Middle Stone Age personal ornaments from Bizmoune Cave, Essaouira, Morocco. *Sci. Adv.* **7**, eabi8620 (2021).

75. C. S. Henshilwood, F. d'Errico, M. Vanhaeren, K. van Niekerk, Z. Jacobs, Middle Stone Age shell beads from South Africa. *Science* **304**, 404 (2004).

76. M. Vanhaeren, L. Wadley, F. d'Errico, Variability in Middle Stone Age symbolic traditions: The marine shell beads from Sibudu Cave, South Africa. *J. Archaeol. Sci. Rep.* **27**, 101893 (2019).

77. J. Zilhão, The emergence of ornaments and art: An archaeological perspective on the origins of "behavioral modernity". *J. Archaeol. Res.* **15**, 1–54 (2007).

78. S. L. Kuhn, M. C. Stiner, E. Güleç, I. Özer, H. Yılmaz, I. Baykara, A. Açıkkol, P. Goldberg, K. Martínez Molina, E. Ünay, F. Suata-Alpaslan, The early Upper Paleolithic occupations at Üçağızlı Cave (Hatay, Turkey). *J. Hum. Evol.* **56**, 87–113 (2009).

79. A. Y. Fedorchenko, N. E. Belousova, Chronology and cultural attribution of the earliest Upper Palaeolithic bone needles of Siberia. *Strat. Plus* **2021**, 217–257 (2021).

80. E. Trinkaus, A. P. Buzhilova, M. B. Mednikova, M. V. Dobrovolskaya, *The People of Sunghir: Burials, Bodies, and Behavior in the Earlier Upper Paleolithic* (Oxford Univ. Press, 2014).

81. Y. V. Kuzmin, M. Boudin, M. Wojcieszak, A. Zazzo, L. van der Sluis, D. I. Stulova, K. N. Gavrilov, E. V. Veselovskaya, S. V. Vasilyev, Sungir revisited: New data on chronology and stratigraphy of the key Upper Paleolithic site, central Russian Plain. *Radiocarbon* **64**, 949–968 (2022).

82. Y. Wei, F. d'Errico, M. Vanhaeren, F. Li, X. Gao, An early instance of Upper Palaeolithic personal ornamentation from China: The freshwater shell bead from Shuidonggou 2. *PLOS ONE* **11**, 1155847 (2016).

83. M. C. Langley, S. O'Connor, E. Piotto, 42,000-year-old worked and pigment-stained Nautilus shell from Jerimalai (Timor-Leste): Evidence for an early coastal adaptation in ISEA. *J. Hum. Evol.* **97**, 1–16 (2016).

84. M. C. Langley, S. Kealy, Mahirta, S. O'Connor, Sequins from the sea: *Nautilus* shell bead technology at Makpan, Alor Island, Indonesia. *Antiquity* **97**, 810–828 (2023).

85. K. Morse, Shell beads from Mandu Mandu Creek rock-shelter, Cape Range peninsula, Western Australia, dated before 30,000 b.p. *Antiquity* **67**, 877–883 (1993).

86. J. Balme, S. O'Connor, Bead making in Aboriginal Australia from the deep past to European arrival: Materials, methods, and meanings. *PaleoAnthropology* **2019**, 177–195 (2019).

87. F. d'Errico, C. S. Henshilwood, Additional evidence for bone technology in the southern African Middle Stone Age. *J. Hum. Evol.* **52**, 142–163 (2007).

88. L. Backwell, F. d'Errico, L. Wadley, Middle Stone Age bone tools from the Howiesons Poort layers, Sibudu Cave, South Africa. *J. Archaeol. Sci.* **35**, 1566–1580 (2008).

89. F. d'Errico, L. Backwell, P. Villa, I. Degano, J. J. Lucejko, M. K. Bamford, T. F. G. Higham, M. P. Colombini, P. B. Beaumont, Early evidence of San material culture represented by organic artifacts from Border Cave, South Africa. *Proc. Natl. Acad. Sci. U.S.A.* **109**, 13214–13219 (2012b).

90. C. S. Henshilwoood, F. d'Errico, C. W. Marean, R. G. Milo, R. Yates, An early bone tool industry from the Middle Stone Age at Blombos Cave, South Africa: Implications for the origins of modern human behaviour, symbolism and language. *J. Hum. Evol.* **41**, 631–678 (2001).

91. T. Higham, R. Jacobi, M. Julien, F. David, L. Basell, R. Wood, W. Davies, C. Bronk Ramsey, Chronology of the Grotte du Renne (France) and implications for the context of ornaments and human remains within the Châtelperronian. *Proc. Natl. Acad. Sci. U.S.A.* **107**, 20234–20239 (2010).

92. A. Picin, D. Stefański, M. Cieśla, P. Valde-Nowak, The beginning of the early Upper Paleolithic in Poland. *J. Paleolit. Archaeol.* **6**, 12 (2023).

93. F. d'Errico, V. Borgia, A. Ronchitelli, Uluzzian bone technology and its implications for the origin of behavioural modernity. *Quat. Int.* **259**, 59–71 (2012).

94. E. M. Wild, M. Teschler-Nicola, W. Kutschera, P. Steier, E. Trinkaus, W. Wanek, Direct dating of early Upper Palaeolithic human remains from Mladeč. *Nature* **435**, 332–335 (2005).

95. M. Otte, F. Biglari, D. Flas, S. Shidrang, N. Zwyns, M. Mashkour, R. Naderi, A. Mohaseb, N. Hashemi, J. Darvish, V. Radu, The Aurignacian in the Zagros region: New research at Yafteh Cave, Lorestan, Iran. *Antiquity* **81**, 82–96 (2007).

96. O. Bar-Yosef, B. Arensburg, A. Belfer-Cohen, P. Goldberg, L. Meignen, M. C. Stiner, N. D. Munro, S. Weiner, in *Quaternary of the Levant: Environments, Climate Change, and Humans*, Y. Enzel, O. Bar-Yosef, Eds. (Cambridge Univ. Press, 2017), pp. 231–240.

97. S. G. Vasiliev, E. P. Rybin, Tolbaga: Upper Paleolithic settlement patterns in the Trans-Baikal region. *Archaeol. Ethnol. Anthropol. Eurasia* **37**, 13–34 (2009).

98. V. I. Tashak, Y. E. Antonova, Palaeolithic complexes of the Podzvonkaya site in the south of western Transbaikal: Comparative characteristic. *Theory Pract. Archaeol. Res.* **28**, 80–96 (2019).

99. M. V. Anikovich, A. A. Sinitsyn, J. F. Hoffecker, V. T. Holliday, V. V. Popov, S. N. Lisitsyn, S. L. Forman, G. M. Levkovskaya, G. A. Pospelova, I. E. Kux'mina, N. D. Burova, P. Goldberg, R. I. Macphail, B. Giaccio, N. D. Praslov, Early upper paleolithic in eastern Europe and implications for the dispersal of modern humans. *Science* **315**, 223–226 (2007).

100. Y. V. Kuzmin, V. S. Slavinsky, A. A. Tsybankov, S. G. Keates. Denisovans, Neanderthals, and early modern humans: A review of the Pleistocene hominin fossils from the Altai Mountains (Southern Siberia). *J. Archaeol. Res.* **30**, 321–369 (2022).

101. E. Trinkaus, A. P. Buzhilova, Diversity and differential disposal of the dead at Sunghir. *Antiquity* **92**, 7–21 (2018).

102. S. Zhang, F. d'Errico, L. R. Backwell, Y. Zhang, F. Chen, X. Gao, Ma'anshan cave and the origin of bone tool technology in China. *J. Archaeol. Sci.* **65**, 57–69 (2016).

103. S. Du, X. Li, L. Zhou, H. Pang, O. Bar-Yosef, X. Wu, Longquan Cave: An early Upper Palaeolithic site in Henan Province, China. *Antiquity* **90**, 876–893 (2016).

104. Y. Wang, X. Zhang, X. Sun, S. Yi, K. Min, D. Liu, W. Yan, H. Cai, X. Wang, D. Curnoe, H. Lu, A new chronological framework for Chuandong Cave and its implications for the appearance of modern humans in southern China. *J. Hum. Evol.* **178**, 103344 (2023).

105. Y. Zhang, X. Wu, S. Zhang, A study of the bone awl from the Ziyang Man site, Sichuan Province. *Acta Anthropol. Sin.* **42**, 1–14 (2023).

106. G. Hamm, P. Mitchell, L. G. Arnold, G. J. Prideaux, D. Questiaux, N. A. Spooner, V. A. Levchenko, E. C. Foley, T. H. Worthy, B. Stephenson, V. Coulthard, C. Coulthard, S. Wilton, D. Johnston, Cultural innovation and megafauna interaction in the early settlement of arid Australia. *Nature* **539**, 280–283 (2016).

107. C. E. Dortch, J. Dortch, Review of Devil's Lair artefact classification and radiocarbon chronology. *Aust. Archaeol.* **43**, 28–32 (1996).

108. R. Cosgrove, Forty-two degrees south: The archaeology of late Pleistocene Tasmania. *J. World Prehist.* **13**, 357–402 (1999).

109. J. Flood, Pleistocene man at Cloggs Cave: His tool kit and environment. *Mankind* **9**, 175–188 (1974).

110. P. Ossa, B. Marshall, C. Webb, New Guinea II Cave: A Pleistocene site on the Snowy River, Victoria. *Archaeol. Ocean.* **30**, 22–35 (1995).

111. L. Doyon, T. Faure, M. Sanz, J. Daura, L. Cassard, F. d'Errico, A 39,600-year-old leather punch board from Canyars, Gavà, Spain. *Sci. Adv.* **9**, eadg083 (2023).

112. A. Calvo, U. Perales, M. García-Rojas, C. Normand, A. Arrizabalaga, Just before sewing needles: A functional hypothesis for Gravettian Noailles-type burins from Isturitz cave (Basque Country, southwestern France). *J. Archaeol. Sci. Rep.* **25**, 420–432 (2019).

113. A. P. Derevianko, M. V. Shunkov, M. B. Kozlikin, A. Y. Fedorchenko, G. D. Pavlenok, N. E. Belousova, in *Problems of Archaeology, Ethnography, Anthropology of Siberia and Neighboring Territories, Vol.* ***XXII***, A. P. Derevianko, Ed. (Institute of Archaeology & Ethnography Press, 2016), pp. 72–75.

114. Y. V. Kuzmin, S. G. Keates, The chronology of hominin fossils from the Altai Mountains, Siberia: An alternative view. *J. Hum. Evol.* **146**, 102834 (2020).

115. L. V. Golovanova, V. B. Doronichev, N. E. Cleghorn, The emergence of bone-working and ornamental art in the Caucasian Upper Palaeolithic. *Antiquity* **84**, 299–320 (2010).

116. O. Bar-Yosef, A. Belfer-Cohen, T. Mesheviliani, N. Jakeli, G. Bar-Oz, E. Boaretto, P. Goldberg, E. Kvavadze, Z. Matskevich, Dzudzuana: An Upper Palaeolithic cave site in the Caucasus foothills (Georgia). *Antiquity* **85**, 331–349 (2011).

117. Y. V. Kuzmin, P. A. Kosintsev, A. D. Stepanov, G. G. Boeskorov, R. J. Cruz, Chronology and faunal remains of the Khayrgas Cave (eastern Siberia, Russia). *Radiocarbon* **59**, 575–582 (2017).

118. W.-W. Huang, Z.-H. Zhang, R.-Y. Fu, B.-F. Chen, J.-Y. Liu, M.-Y. Zhu, H.-K. Wu, Bone artefacts and ornaments from Xiaogushan site of Haicheng, Liaoning Province. *Acta Anthropol. Sin.* **1986**, 259–266 (1986).

119. J.-F. Zhang, W.-W. Huang, B.-Y. Yuan, R.-Y. Fu, L.-P. Zhou, Optically stimulated luminescence dating of cave deposits at the Xiaogushan prehistoric site, northeastern China. *J. Hum. Evol.* **59**, 514–524 (2010).

120. L. Doyon, Bone needles in China and their implications for late Pleistocene hominin dispersals. *Acta Anthropol. Sin.* **38**, 362–372 (2019).

121. W. Wang, C. Bae, A. Xu, Chinese prehistoric eyed bone needles: A review and assessment. *J. World Prehist.* **33**, 385–423 (2020).

122. S.-X. Yang, J.-F. Zhang, J.-P. Yue, R. Wood, Y.-J. Guo, H. Wang, W.-G. Luo, Y. Zhang, E. Raguin, K.-L. Zhao, Y.-X. Zhang, F.-X. Huan, Y.-M. Hou, W.-W. Huang, Y.-R. Wang, J.-M. Shi, B.-Y. Yuan, A. Ollé, A. Queffelec, L.-P. Zhou, C.-L. Deng, F. d'Errico, M. Petraglia. Initial Upper Palaeolithic material culture by 45,000 years ago at Shiyu in northern China. *Nat. Ecol. Evol.* **8**, 552–563 (2024).

123. F. Li, F.-Y. Chen, X. Gao, "Modern behaviors" of ancient populations at Shuidonggou Locality 2 and their implications. *Quat. Int.* **347**, 66–73 (2014).

124. S. G. Keates, Y. V. Kuzmin, Shuidonggou localities 1 and 2 in northern China: Archaeology and chronology of the Initial Upper Palaeolithic in north-east Asia. *Antiquity* **89**, 714–720 (2015).

125. Y. Song, X. Li, X. Wu, E. Kvavadze, P. Goldberg, O. Bar-Yosef, Bone needle fragment in LGM from the Shizitan site (China): Archaeological evidence and experimental study. *Quat. Int.* **400**, 140–148 (2016).

126. F. Li, C. J. Bae, C. Bronk Ramsey, F. Chen, X. Gao, Re-dating Zhoukoudian Upper Cave, northern China and its regional significance. *J. Hum. Evol.* **121**, 170–177 (2018).

127. B. A. Odar, A Dufour bladelet from Potočka zijalka (Slovenia). *Archeol. Vestn.* **59**, 9–16 (2008).

128. M. Anghelinu, M. Mărgărit, L. A. Niţă, A paleolithic eyed needle from Bistricioara-Lutărie III (Ceahlău Basin, northeastern Romania). *Stud. Preist.* **14**, 27–35 (2017).

129. A. Calvo, A. Arrizabalaga, Piecing together a new mosaic: Gravettian lithic resources and economic territories in the western Pyrenees. *Archaeol. Anthropol. Sci.* **12**, 282 (2020).

130. J. Riel-Salvatore, C. Gravel-Miguel, in *The Oxford Handbook of the Archaeology of Death and Burial*, S. Tarlow, L. Nilsson Stutz, Eds. (Oxford Univ. Press, 2013), pp. 303–346.

131. F. d'Errico, M. Vanhaeren, in *Death Shall Have No Dominion*, C. Renfrew, M. J. Boyd, I. Morley, Eds. (Cambridge Univ. Press, 2015), pp. 45–61.

132. L. Ledoux, G. Berillon, N. Fourment, X. Muth, J. Jaubert, Evidence of the use of soft footwear in the Gravettian cave of Cussac (Dordogne, France). *Sci. Rep.* **11**, 22727 (2021).

133. J. F. Hoffecker, *Desolate Landscapes: Ice-Age Settlement in Eastern Europe* (Rutgers Univ. Press, 2002).

134. L. G. Straus, A quarter-century of research on the Solutrean of Vasco-Cantabria, Iberia and beyond. *J. Anthropol. Res.* **56**, 39–58 (2000).

135. G. Ramstein, M. Kageyama, J. Guiot, H. Wu, C. Hély, G. Krinner, S. Brewer, How cold was Europe at the Last Glacial Maximum? A synthesis of the progress achieved since the first PMIP model-data comparison. *Clim. Past* **3**, 331–339 (2007).

136. R. F. Rifkin, Assessing the efficacy of red ochre as a prehistoric hide tanning ingredient. *J. Afr. Archaeol.* **9**, 131–158 (2011).

137. K. MacDonald, Fire-free hominin strategies for coping with cool winter temperatures in north-western Europe from before 800,000 to circa 400,000 years ago. *PaleoAnthropology* **7**, 7–26 (2018).

138. R. Hosfield, *The Earliest Europeans: A Year in the Life—Seasonal Survival Strategies in the Lower Palaeolithic* (Oxbow Books, 2020).

139. L. R. Binford, *Constructing Frames of Reference: An Analytical Method for Archaeological Theory Building using Ethnographic and Environmental Data Bases* (*University of California Press*, 2001).

140. R. L. Kelly, *The Lifeways of Hunter-Gatherers: The Foraging Spectrum* (Cambridge Univ. Press, 2013).

141. K. Sterelny, Ethnography, archaeology, and the late Pleistocene. *Philos. Sci.* **89**, 415–433 (2022).

142. L. A. Otak, in *Arctic Clothing of North America—Alaska, Canada, Greenland*, J. C. H. King, B. Pauksztat, R. Storrie, Eds. (McGill-Queen's Univ. Press, 2005), pp. 74–79.

143. M. Kodama, in *Ainu: Spirit of a Northern People*, W. W. Fitzhugh, C. O. Dubreuil, Eds. (Smithsonian Institution, 1999), pp. 313–326.

144. S. K. Lothrup, *The Indians of Tierra del Fuego* (Museum of the American Indian, 1928).

145. M. Gusinde, *The Lost Tribes of Tierra del Fuego: Selk'nam, Yamana, Kawésqar* (Thames and Hudson, 2015).

146. C. R. Darwin, *Journal of Researches into the Geology and Natural History of the Various Countries Visited by H.M.S. Beagle, Under the Command of Captain Fitzroy, R.N. from 1832 to 1836* (Henry Colburn, 1839).

147. I. Gilligan, *Another Tasmanian Paradox: Clothing and Thermal Adaptations in Aboriginal Australia* (Archaeopress, 2007).

148. I. Gilligan, Clothing and climate in Aboriginal Australia. *Curr. Anthropol.* **49**, 487–495 (2008).

149. R. Cosgrove, A. Pike-Tay, W. Roebroeks, in *Southern Asia, Australia and the Search for Human Origins*, R. Dennell, M. Porr, Eds. (Cambridge Univ. Press, 2014), pp. 175–188.

150. M. F. Péron, *A Voyage of Discovery to the Southern Hemisphere, Performed by Order of the Emperor Napoleon, During the Years 1801, 1802, 1803, and 1804* (Richard Phillips, 1809).
